# Research on the Thermal Aging Mechanism of Polyvinyl Alcohol Hydrogel

**DOI:** 10.3390/polym16172486

**Published:** 2024-08-31

**Authors:** Chunkun Chen, Xiangyang Liu, Jiangtao Wang, Haoran Guo, Yingjun Chen, Ningfei Wang

**Affiliations:** School of Aerospace Engineering, Beijing Institute of Technology, Beijing 100081, China; ckchen@bit.edu.cn (C.C.); wangjiangtao@bit.edu.cn (J.W.); 3120235069@bit.edu.cn (H.G.); 3220230059@bit.edu.cn (Y.C.); wangningfei@bit.edu.cn (N.W.)

**Keywords:** PVA hydrogel, high-temperature accelerated aging, mechanical property, moisture content, hydrogen bonding, crystallinity

## Abstract

Polyvinyl alcohol (PVA) hydrogels find applications in various fields, including machinery and tissue engineering, owing to their exceptional mechanical properties. However, the mechanical properties of PVA hydrogels are subject to alteration due to environmental factors such as temperature, affecting their prolonged utilization. To enhance their lifespan, it is crucial to investigate their aging mechanisms. Using physically cross-linked PVA hydrogels, this study involved high-temperature accelerated aging tests at 60 °C for 80 d and their performance was analyzed through macroscopic mechanics, microscopic morphology, and microanalysis tests. The findings revealed three aging stages, namely, a reduction in free water, a reduction in bound water, and the depletion of bound water, corresponding to volume shrinkage, decreased elongation, and a “tough-brittle” transition. The microscopic aging mechanism was influenced by intermolecular chain spacing, intermolecular hydrogen bonds, and the plasticizing effect of water. In particular, the loss of bound water predominantly affected the lifespan of PVA hydrogel structural components. These findings provide a reference for assessing and improving the lifespan of PVA hydrogels.

## 1. Introduction

Hydrogels are polymer materials dispersed in water [1,2] that possess properties such as flexibility, lubricity, conductivity, and biocompatibility. They are widely used in aerospace [3,4], machinery [5], energy [6,7], medicine [8,9], and agriculture [10,11]. Among the various hydrogels, polyvinyl alcohol (PVA) hydrogels, formed by the physical cross-linking of PVA molecules, exhibit good mechanical properties [12] and are suitable for load-bearing structural components [13,14,15]. Lifespan, which is crucial for structural components, depends on the quasi-static mechanical property changes related to physical and chemical aging mechanisms under storage and usage conditions. As a material with high water content, hydrogels exhibit significant changes in mechanical properties over prolonged periods of load and storage due to aging behaviors such as dehydration [16], resulting in their inability to meet the expected service lifespan requirements, which largely restricts their use [17,18]. To enhance the lifespan of PVA hydrogels, it is necessary to investigate their aging behaviors, clarify the fundamental mechanisms limiting their lifespan based on the specific aging process, and take targeted measures to slow down the aging process.

High-temperature accelerated aging testing is a typical method for investigating the long-term storage and usage performances of polymer materials [19]. Tang et al. [20] studied the thermal aging behavior of tightly wrapped PVA hydrogels at 40–70 °C for 360 d and found that the mechanical properties hardly changed during the thermal aging process. After 360 d of aging, mechanical testing became difficult due to sample dehydration, yet no in-depth study was conducted on the storage process after water loss in the PVA hydrogels. Additionally, no other research has focused on the high-temperature accelerated aging testing of PVA hydrogels. However, research on PVA hydrogel annealing tests, which are similar to high-temperature accelerated aging tests, can serve as a useful reference. Cao et al. [21] annealed PVA hydrogel at 50 °C for 8 h and found that the annealing process enhanced intermolecular hydrogen bonding, increased crystallinity and crystal size, and formed a dense hydrogel with a well-defined crystal structure, leading to an increased tensile strength. According to Chen et al. [22], annealing PVA hydrogels at 100 °C for 1 h could effectively increase their crystallinity, thereby improving their tensile strength, maximum elongation, and other mechanical properties. However, the duration of high-temperature accelerated aging tests, usually equivalent to long-term usage, is much longer than the annealing time. The above studies did not determine the degradation pattern of the mechanical properties of PVA hydrogels throughout their entire lifecycle.

Additional research on the aging of different hydrogels and comparable polymer materials has provided insights into the aging mechanism of PVA hydrogels. Xiong et al. [23] found that polyacrylamide hydrogels undergo significant dehydration, volume shrinkage, and molecular chain breakage after aging at 150 °C for 12 h, leading to poor thermal stability. Law et al. [24] investigated the impact of high-temperature aging at 90 and 140 °C for 12 d on the microstructural changes of polyacrylamide materials, revealing an increase in crystallinity owing to aging. Sližová et al. [25] studied the aging characteristics of polyacrylamide materials in long-term storage over 30 y, revealing a sharp decrease in maximum elongation and a significant increase in crystallinity after prolonged storage. Based on these studies, it is inferred that PVA hydrogels, as semi-crystalline hydrogel materials, may undergo aging phenomena such as dehydration, volume shrinkage, and changes in crystallinity during the aging process, leading to a decrease in their mechanical properties.

In studies of the mechanical property regulation of PVA hydrogels, researchers have typically explored the correlation between mechanical properties and microscale parameters, such as hydrogen bonds and crystallinity, which have a certain reference value for investigating the aging mechanism of mechanical properties. Hodge et al. [26] found that breaking the intermolecular hydrogen bonds of PVA by combining it with water increased the free volume and mobility of PVA molecular chains, thus enhancing the mechanical properties of PVA. Briscoe et al. [27] and Li et al. [28] demonstrated that the viscoelasticity of PVA hydrogels was primarily determined by hydrogen-bond interactions. The attractive force of hydrogen bonds promoted ordered molecular arrangement, altering the crystallization behavior and cross-linked network structure of PVA hydrogels, and improving the mechanical properties, such as tensile strength [29]. Li et al. [30] found that completely dehydrated PVA hydrogels lost their viscoelasticity and exhibited apparent brittleness. This indicates that water has a significant impact on the mechanical properties of PVA hydrogels, which requires special attention in aging studies.

Therefore, conducting high-temperature accelerated aging experiments on PVA hydrogels is essential to study the evolution of their mechanical properties and aging mechanisms. Using physically cross-linked PVA hydrogels, this study conducted high-temperature accelerated aging tests at 60 °C for 80 d, along with macroscopic mechanics, microscopic morphology, and microstructural analysis tests, to elucidate the mechanisms of material aging while establishing correlations between macroscopic mechanical properties and microscopic phenomena.

## 2. Materials and Methods

### 2.1. Sample Preparation

**Figure 1:**
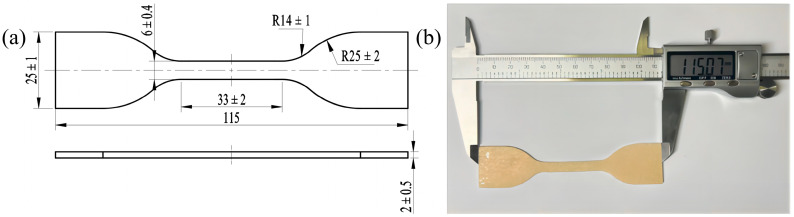
Experimental sample: (**a**) dimensions; (**b**) actual sample.

The PVA hydrogel materials used in this study were primarily formed through physical cross-linking, such as hydrogen bonding [12], and were obtained from the Lanzhou Institute of Chemical Physics, Chinese Academy of Sciences. First, the PVA sol was dried at room temperature to obtain a dry PVA gel. Subsequently, it was immersed in a salt solution to form a dense hydrogen bond network between the hydroxyl groups of the PVA molecular chains, which promoted the aggregation and crystallization of the PVA molecular chains, yielding a salt-induced PVA hydrogel with a high degree of crystallinity. The salt-induced PVA hydrogel was dialyzed with deionized water to obtain the final PVA hydrogel. By adjusting factors such as the PVA mass fraction, salting-out concentration, experimental temperature, and time, the properties of PVA hydrogels can be effectively regulated. The initial maximum tensile strength of the customized PVA hydrogel prepared and used in this study was approximately 5 MPa, with a maximum elongation of around 300%, a moisture content of about 62%, and a polymerization degree of 1750 ± 50. The PVA hydrogel samples were cut to obtain aging samples (dog bones, 115 mm × 25 mm × 2 mm), as shown in Figure 1.

### 2.2. High-Temperature Accelerated Aging Test

The aging temperature and time were selected to be 60 °C and 80 d, respectively. The temperature range for the accelerated aging test of polymers is generally between 50 and 120 °C [21]. Due to the rapid loss of moisture at 100 °C, it is difficult to observe effective experimental phenomena. Considering the experimental duration, this study selected an aging temperature of 60 °C. The selected accelerated aging temperature was suitable and feasible.

**Figure 2:**
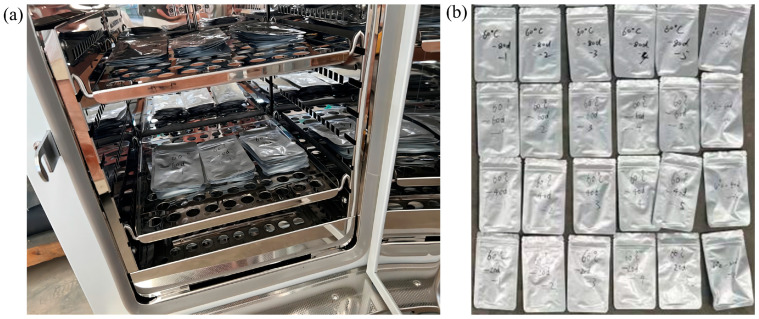
High-temperature accelerated aging test: (**a**) equipment; (**b**) sample classification.

In order to better observe the experimental phenomenon and distinguish the aging stages more reasonably, aluminum bags were used to provide a semi-sealed environment to appropriately slow down the loss of moisture [19,31,32]. The PVA hydrogel materials wrapped in aluminum bags were subjected to high-temperature accelerated aging tests in a WGL-45L high-temperature test chamber. The equipment used in the test chamber is illustrated in Figure 2a. During the aging process, we changed the placement of the samples every 10 days to minimize the impact of their positioning on the results. To monitor the changes in sample performance with aging time, samples were taken after 20, 40, 60, and 80 d of aging, with six samples collected at each sampling time point, as shown in Figure 2b.

### 2.3. Characterization

Based on the physically cross-linked PVA hydrogels, we focused on conducting tests that characterize the physical cross-linking conditions, such as water content, hydrogen bond changes, and crystallinity. Additionally, we performed mechanical tests, hardness tests, and SEM analyses to evaluate changes in the mechanical properties, establishing relevant correlations.

The aged samples were directly subjected to moisture content and mechanical testing. The moisture content of the aged samples was measured using a rapid moisture analyzer (DHS-20A), with a weighing accuracy of ±0.001 g. The other five samples were subjected to uniaxial tensile tests using an SUST CMT2502 universal material testing machine at room temperature, with a stretching rate of 100 mm/min and a displacement resolution of 2 μm. Considering the dimensional changes caused by the shrinkage of the samples during aging, we measured the size parameters of the material before each mechanical test and incorporated these measurements into our calculations, thereby obtaining variations in the mechanical performance.

Nanoindentation tests were performed using a nanoindentation instrument (NANOLNDENTER G200) to determine the hardness of the sample during the aging process, with a displacement resolution of 0.02 nm, a maximum depth of 2 μm, and a strain rate of 0.05 s^−1^. To eliminate the effect between indentation areas, each indentation point was at least 20 µm apart. The samples were tested five times at each aging time point.

The fractured cross-sections of the samples after mechanical testing were observed at room temperature using a scanning electron microscope (FEI QUANTA 600F SEM). SEM images were captured at each aging time point to observe the behavior of the samples during the aging process.

Attenuated total reflection Fourier transform infrared spectroscopy (ATR-FTIR), X-ray diffraction (XRD), and differential scanning calorimetry (DSC) analyses were performed on the fractured specimens after the mechanical testing. All performance tests were conducted by sampling and testing three different areas of the same specimen to ensure accuracy and reproducibility.

An ATR-FTIR spectrometer (INVENIO-S) was used to track the changes in hydrogen bonds mediated by hydroxyl groups (-OH) during aging, with a spectral range of 4000–400 cm^−1^ and an instrument resolution of 4 cm^−1^. The ATR-FTIR spectroscopy used a Ge prism, carrying out 32 scans, and the angle of incidence of the incident beam was 45°.

In order to study the changes in the crystallization behavior of the samples during the aging process, measurements by XRD were carried out over the 2θ range from 5° to 60° at a scanning rate of 20°/min using an X-ray diffractometer (BRUKER D8 AD-VANCE), with Cu Ka radiation (λ = 1.54184 Å).

The thermal transformation and crystallization behavior of the hydrogel were studied using a differential scanning calorimeter (NETZSCH DSC 214 POLYMA), with a heating rate of 10 °C/min up to 300 °C. In the experiment, the materials were prepared as samples weighing about 15 mg, with a nitrogen flow rate of 50 mL/min utilized as the shielding and purging gas.

## 3. Results

### 3.1. Macro Performance Analysis

**Figure 3:**
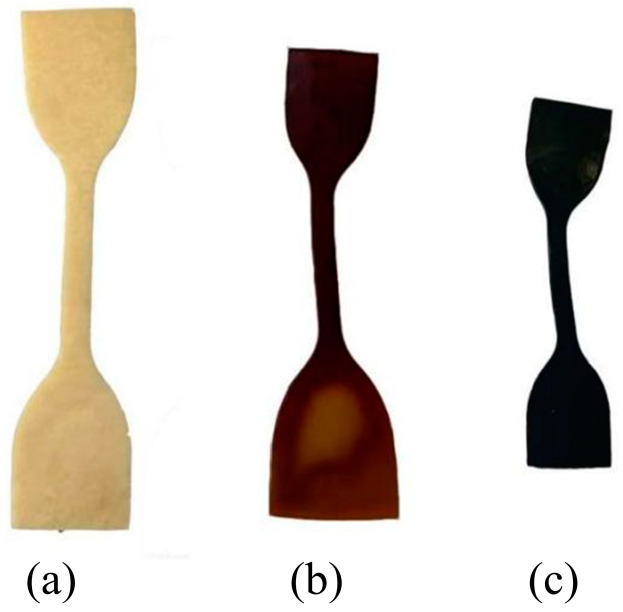
Macroscopic performance changes of samples during aging process: (**a**) unaged sample; (**b**) sample after 40 d of aging; (**c**) sample after 80 d of aging.

Figure 3 shows images of the PVA hydrogels at different aging times. As aging progressed, the color of the PVA hydrogel changed from milky white to brown and gradually to dark black. Simultaneously, the samples underwent apparent deformation and warping, resulting in overall volume shrinkage. Volume changes of samples during the aging process were estimated, as shown in Table 1. Significant shrinkage occurred in the sample volume during the aging process from 0 to 20 d. With aging from 20 to 60 d, the shrinkage of the sample volume tended to decrease. After 60 d, the volume shrinkage rate increased slightly.

The variation in the moisture content of the samples exhibited similar patterns. As aging progressed, the surface-wet hydrogels became dry and smooth. The moisture in the samples gradually decreased, with an almost complete loss of water after 80 days of aging. The semi-sealed PVA hydrogel exhibited a significant water loss behavior during the aging process, and water was lost to the environment through evaporation.

### 3.2. ATR-FTIR Results

**Figure 4:**
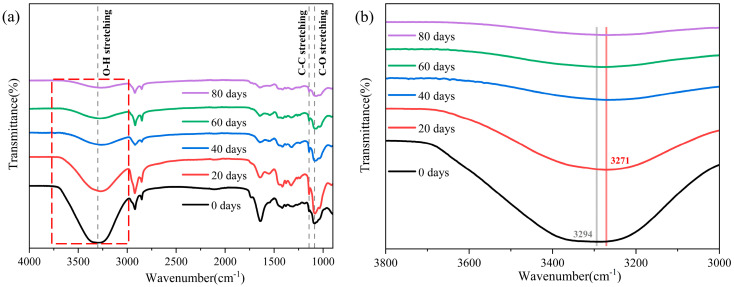
Changes in microscopic functional groups during aging process: (**a**) ATR-FTIR spectroscopic analysis; (**b**) spectrum of hydroxyl changes during aging process.

Changes in the microscopic functional groups of the PVA hydrogels before and after aging at 60 °C were determined using an ATR-FTIR spectrometer, demonstrating the impact of moisture loss from a microscopic perspective, as shown in Figure 4a. The samples exhibited seven absorption peaks at 3300, 2925, 2852, 1640, 1422, 1143, and 1087 cm^−1^. The peak at 3300 cm^−1^ corresponded to the hydroxyl (-OH) stretching vibration, whereas the peaks at 2925 and 2852 cm^−1^ were primarily attributed to the asymmetric and symmetric stretching vibrations of the methylene group (-CH_2_). The peak at 1640 cm^−1^ corresponded to the double-bond stretching vibrations of carbonyl (C=O), and the peak at 1422 cm^−1^ was attributed to the stretching vibrations of ethyl (-CH) of PVA. In addition, the characteristic peak at 1143 cm^−1^ was attributed to the C-C stretching vibration associated with the C-O stretching vibration, which was attributed to the crystallinity within the PVA hydrogel networks [12,33], confirming the crystallization behavior of the PVA hydrogel.

In the aging process, we primarily focused on the changes in hydrogen bonds because it is the primary cross-linking method for PVA hydrogels. The mechanical properties of the PVA hydrogels were significantly affected by the interactions between hydrogen bonds [28,30]. As shown in Figure 4b, the overall peak of the -OH stretching vibration band decreased owing to the decrease in water content during the aging process. The absorption band in the range of 3000–3650 cm^−1^ is composed of the hydrogen bond -OH stretching vibration band (3000–3600 cm^−1^) and free -OH stretching vibration band (3600–3650 cm^−1^). This wider absorption band is produced by the stretching of the bonded -OH groups (forming intermolecular and intramolecular hydrogen bonds) and non-bonded free -OH groups together [34,35]. Similar to the phenomenon observed by Mandal et al. [36], the width of the absorption band of the free -OH stretching vibration in the sample decreased significantly as aging progressed, indicating a considerable reduction in free hydroxyl groups during the aging process. Simultaneously, the peak of the absorption band caused by hydroxyl stretching shifted from 3294 to 3271 cm^−1^. This phenomenon proves that stronger intermolecular hydrogen bonds are formed between the PVA chains owing to aging [37,38]. This phenomenon was also confirmed by Li et al. [30]. As a semi-crystalline polymer, PVA hydrogels consist of a water phase, an amorphous PVA phase, and a crystalline PVA phase [39]. The enhancement of intermolecular hydrogen bonding promotes the ordered arrangement of molecular chains, thereby altering the crystalline behavior and crosslinking network structure of PVA hydrogels, which, in turn, affects their mechanical properties. Furthermore, this shift toward a lower frequency (red shift) indicates that the strength of the -OH group decreased with an increasing aging time [40]. This result could be attributed to the fact that the increase in intermolecular hydrogen bonds caused the -OH groups to become more relaxed, which provided a basis for other changes in aging performance.

### 3.3. Thermal Analysis Results

**Figure 5:**
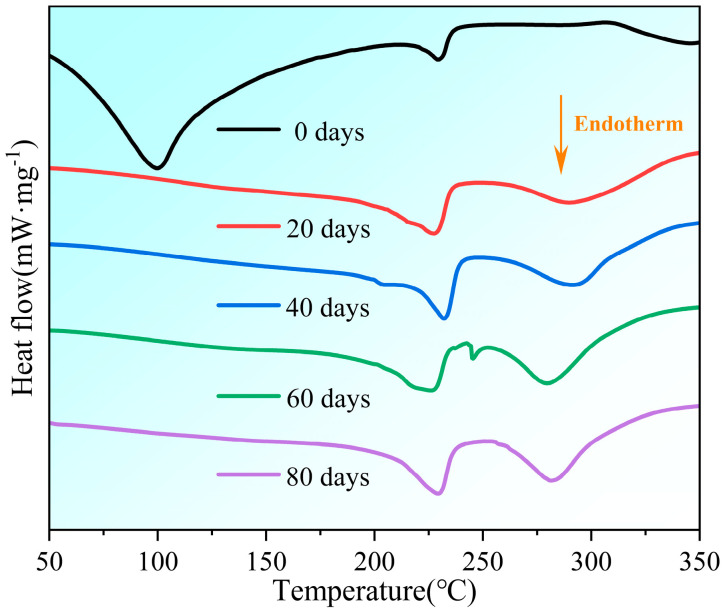
Changes in differential scanning calorimetry analysis of samples during aging process.

Changes in the crystallinity of the samples during aging were obtained by the thermal analysis test, and the results are shown in Figure 5.

The initial endothermic peak of the unaged PVA hydrogel was observed at a temperature below 100 °C, indicating the quick evaporation of free water during the initial phases of the aging process. The peak value decreased significantly at other aging times, combining the phenomenon of continued water loss, as shown in Table 1. This indicated that most of the water lost after 20 d was bound water. The endothermic peak that appeared in the temperature range of 200–240 °C was attributed to the melting of semicrystalline PVA [12]. Crystallinity can be calculated from the endothermic peak of PVA melting using the following expression:

(1) Xcrystallinity=ΔHmΔHm*

where Δ*H_m_* is the enthalpy of fusion of the experimental material and ΔH_m_* is the enthalpy of fusion of 100% crystalline PVA, taken as 138.6 J/g [41].

The crystallinity of the PVA material exhibited an increasing trend followed by fluctuations with aging, as shown in Table 2. This phenomenon was observed in a study by Lee et al. [42] and confirmed by the XRD pattern.

Simultaneously, with the aging of the PVA hydrogels, there was a shift in the thermal decomposition peak from around 350 °C to 290 °C, showing a decline in the thermal stability of the aged materials. The presence of this peak is mostly caused by the elimination process of -OH groups on the main chain of PVA molecules during the temperature range of 280–350 °C [43]. The ATR-FTIR test results indicated that the -OH peak location underwent a red shift, suggesting a decrease in the strength of the -OH group with an increasing aging time. Heat can easily remove the group at a lower energy, thereby reducing the thermal stability of PVA hydrogels.

### 3.4. XRD Results

**Figure 6:**
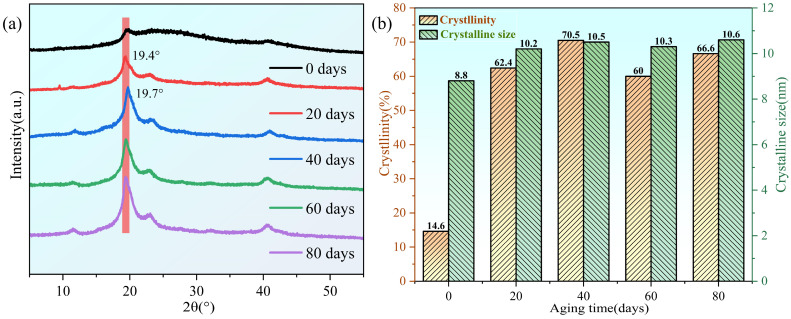
Changes in crystallinity analysis of samples during aging process: (**a**) X-ray diffraction curve; (**b**) changes in crystallinity and average size of crystalline regions during aging process.

Figure 6a shows the XRD pattern of the PVA hydrogel during aging at 60 °C. The sharp diffraction peak at 2*θ* = 19.4° corresponds to the (101) crystal plane of PVA, confirming the semi-crystalline nature of the PVA hydrogel [44], which is attributed to the strong intermolecular interactions between the PVA molecular chains through hydrogen bonds [45]. As previously mentioned, the presence of the peak at 1143 cm^−1^ in the FTIR spectrum also confirmed the existence of crystallization in the PVA hydrogel. Moreover, the diffraction peak position shifted slightly toward 2*θ* = 19.7°, which was attributed to the reduction in water content during the aging of the PVA hydrogel [33]. According to Bragg’s law, the water content in PVA hydrogels decreases with an increasing aging time, resulting in a decrease in the interplanar spacing and rightward shift of the diffraction peak.

(2) 2dsinθ=nλ

where *n* is an integer multiple of the wavelength, *d* is the distance between parallel crystal planes, *λ* is the wavelength of the incident wave, and *θ* is the angle between the incident light and crystal plane.

During aging, diffraction peaks appeared at 2*θ* = 22.9° and 40.8°, corresponding to the semi-crystalline PVA (200) and (102) planes, respectively [46]. All diffraction peaks revealed an increasing trend and fluctuations in crystallinity with aging time, which was consistent with the DSC results, indicating that the dynamic process favored crystallization during the aging of the PVA hydrogels. The average grain size of the crystals was calculated from the XRD diffraction peaks using the Debye-Scherrer formula [47]:

(3) D=KλBcosθ

where *K* is the Scherrer constant (0.89), *B* is the measured sample diffraction peak width at half maximum, *θ* is the Bragg angle, and *λ* is the X-ray wavelength.

The average size of the crystalline regions increased with an increasing basic stability of the fluctuations, which were 8.8, 10.2, 10.5, 10.3, and 10.6 nm (Figure 6b). The increase in crystallinity and the average size of the crystalline regions during the early stages of aging is primarily attributed to the increased hydrogen bonding between the PVA chains, whereas the later fluctuations reflect the dynamic process of crystalline generation and recombination.

### 3.5. Mechanical Properties

**Figure 7:**
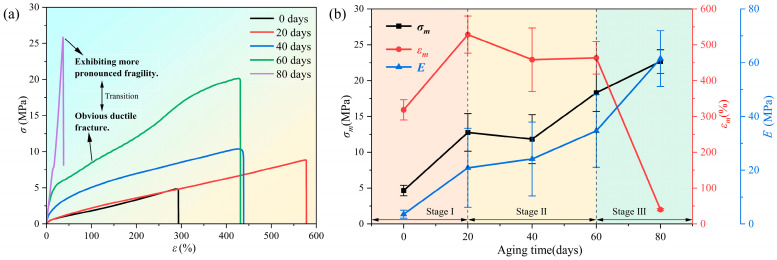
Changes in mechanical properties of samples during aging process: (**a**) stress–strain curve at different aging times; (**b**) changes in maximum tensile strength, maximum elongation, and initial modulus during aging process.

The mechanical property tests demonstrated the impact of the aforementioned changes on the properties of the hydrogel. Typical stress–strain curves of the samples under accelerated aging at high temperatures were obtained by conducting uniaxial tensile tests at room temperature, as shown in Figure 7a. With aging, the shape of the stress–strain curve changes significantly, exhibiting a notable absence of a yield stage in the later stages. This finding indicates a transition of the PVA hydrogel from a tough to a brittle state.

As shown in Figure 7b, maximum elongation can serve as a parameter for characterizing the aging of mechanical properties, and the aging process of the PVA hydrogel can be divided into three stages based on the variations in maximum elongation during aging. During the aging process of 0–20 d, the maximum tensile strength, elongation, and initial modulus of the PVA hydrogel exhibited an increasing trend. With continued aging, the second aging stage occurred between 20 and 60 d, and the specimens exhibited a basic increasing trend in the maximum tensile strength and initial modulus, whereas the maximum elongation exhibited a decreasing trend. This finding indicated that the PVA hydrogel lost its tensile plasticity with aging. The third stage occurred after 60 d of aging, and the specimens gradually exhibited a “glassy” state with greater brittleness. The mechanical properties of the samples degraded with aging, rendering macroscopic mechanical tensile testing challenging. A similar observation was reported by Li et al. [30,35], who prepared PVA hydrogels with various water contents and tested their mechanical properties. The study found that, as the moisture content decreased, both the maximum tensile strength and modulus increased. The elongation initially increased and subsequently decreased, ultimately causing a shift from toughness to brittleness. Nevertheless, as their research directly presupposed the water content of the PVA hydrogels, the effects of water content and mechanical properties changing with aging time could not be determined, thus lacking significance with regard to material aging.

**Figure 8:**
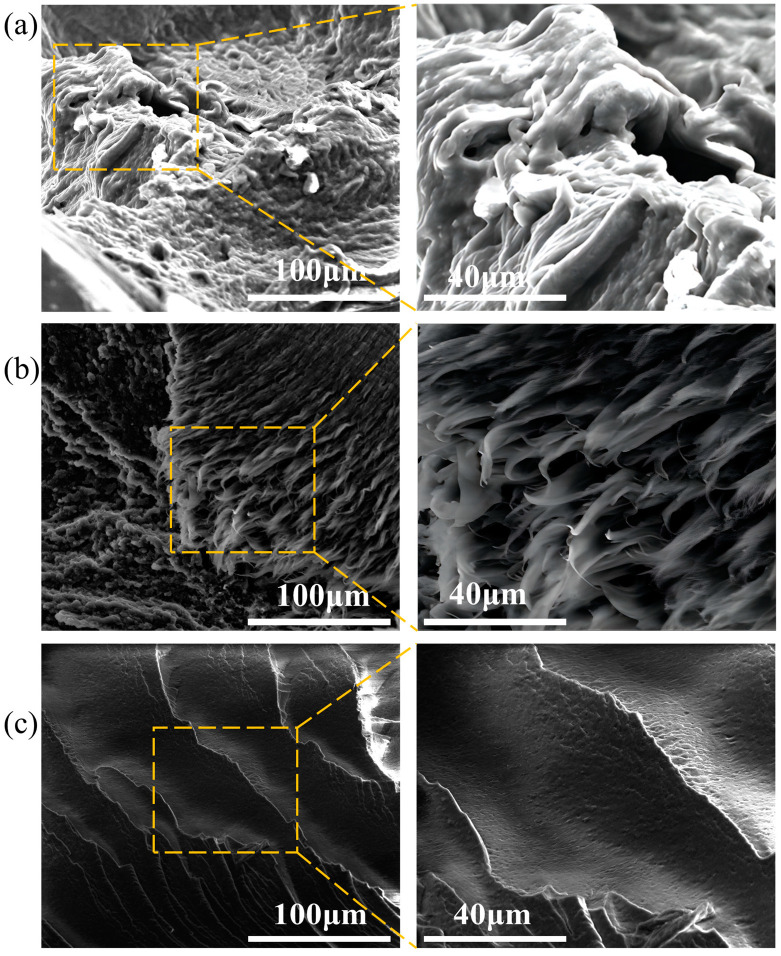
Changes in microscopic cross-section during aging process: (**a**) unaged sample; (**b**) sample after 40 d of aging; (**c**) sample after 80 d of aging.

Figure 8 shows cross-sectional images of the samples aged at 60 °C for 0–80 days under a scanning electron microscope, elucidating the microstructural changes of the PVA hydrogel during the aging process. As shown in Figure 8a, the SEM images indicate that the fracture mode of the unaged sample was ductile, with a rough fracture surface and uneven fracture edges that appeared fibrous. As aging progressed, the fracture mode of the sample gradually transitioned to brittle fracture, with the fracture surface becoming smooth and flat, as shown in Figure 8b,c, respectively. This “ductile-brittle transition” fracture mode corresponds to the analysis of macroscopic mechanical properties.

**Figure 9:**
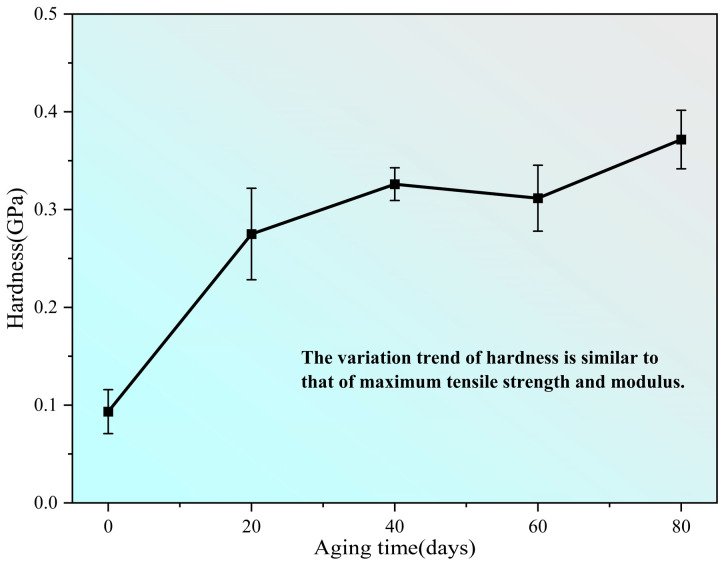
Change in hardness of samples during aging process.

The hardness of the PVA hydrogel was determined in nanoindentation tests revealing a fluctuating increasing trend with continued aging, which is similar to the changing trend of the maximum tensile strength and initial modulus, as shown in Figure 9.

## 4. Discussion

**Figure 10:**
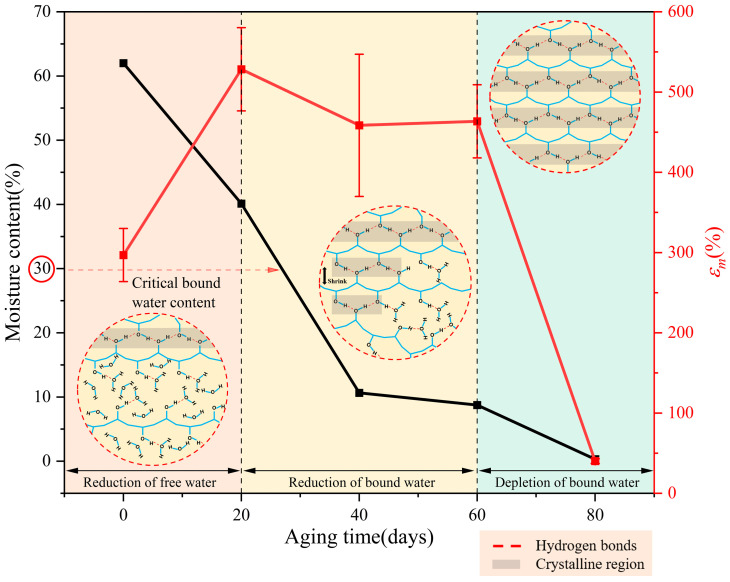
The aging process of PVA hydrogel.

Based on the above results, we analyzed the aging mechanism of the PVA hydrogel. The unaged PVA hydrogel possessed a relatively high water content, with a portion of this water acting as a solvent that expanded the PVA molecular chains, categorized as free water. Additionally, it could function as bound water, forming hydrogen bonds with the -OH groups on the PVA main chains. According to Hodge et al. [26], the critical bound water content at which saturated hydrogen bonding occurred between PVA and water molecules was 30 wt%. Li et al. [30] indicated that bound water primarily acted as a plasticizer, providing sufficient free volume for the movement of PVA molecules, thereby ensuring the flowability of PVA molecular chains. Based on the aging characteristics presented by the PVA hydrogels, combined with the changes in their mechanical properties and water loss characteristics during the aging process, the aging of PVA hydrogels can be basically divided into three stages, namely, the reduction in free water, the reduction in bound water, and the depletion of bound water, as shown in Figure 10.

**Figure 11:**
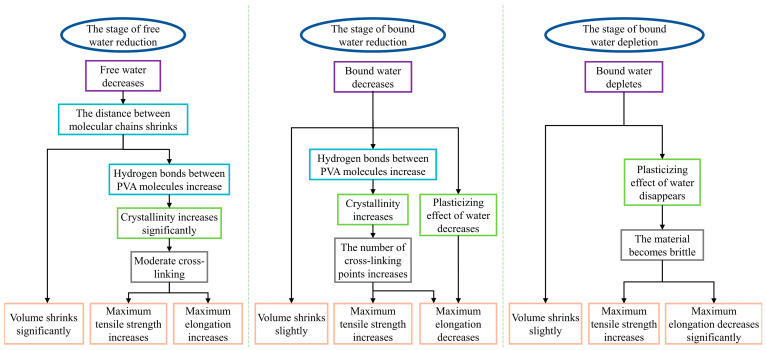
Thermal aging mechanism of PVA hydrogel.

As shown in Figure 10 and Figure 11, most PVA molecular chains were irregularly entangled and formed amorphous regions before aging, owing to their high water content and low crystallinity. The presence of more water reduces the entanglement of PVA molecular chains [48,49], resulting in weaker interactions between them. During the initial aging stage, free water was first lost, as shown by the DSC results. The loss of free water primarily affected the volume of the PVA hydrogel, resulting in a significant shrinkage of the material volume during the early aging stages. As the volume of the PVA hydrogel shrank, the proximity of different PVA molecular chains promoted the formation of hydrogen bonds between the PVA molecules, causing some amorphous regions to transform into ordered crystalline regions, as confirmed by ATR-FTIR and DSC crystallinity calculations. During the aging process, the changes in tensile strength and hardness exhibited similar increasing and fluctuating trends with changes in crystallinity. This result was attributed to the dispersion of the ordered crystalline regions of the PVA hydrogel in the amorphous regions, with the crystalline regions acting as physical cross-linking points, linking the cross-linked network structure to withstand external stress [50]. Consequently, the tensile strength and hardness increased. This mechanism is similar to the reinforcement effect of crystallinity during the annealing process [21,22,51], resulting in enhanced mechanical properties of the hydrogel. However, as previously mentioned, the more critical stages concerning the aging mechanism and lifespan of PVA hydrogels are the next two stages where performance degradation occurs. These two stages were not considered or observed in the annealing experiments. Simultaneously, because free water serves as a lubricant [30], the reduction in water content at this stage does not significantly affect the free volume of the molecular chains, and the plasticizing effect of the bound water provides sufficient free volume for the movement of the PVA molecular chains, thereby ensuring the flowability of the molecular chains. Therefore, in the reduced free water stage, the PVA hydrogel exhibited significant shrinkage in volume, along with an increase in maximum tensile strength and elongation at break.

As the aging progressed and the water content continued to decrease, the decrease in bound water played a dominant role in the second stage of aging. Because of the decrease in bound water, the -OH groups that were originally bound to the water were released. Consequently, the free volume of the PVA molecular chains decreased, and the intermolecular hydrogen bonding interactions increased [30]. The reduction in free volume led to a gradual weakening of the plasticizing effect of water, resulting in a decreased mobility of the PVA molecular chains and a decrease in elongation at break. The increase in intermolecular hydrogen bonding interactions led to an increase in crystallinity, resulting in an increase in the tensile strength. Simultaneously, the increase in crystallinity made the material more brittle owing to the increase in cross-linking points. Therefore, in the reduced bound water stage, the PVA hydrogel exhibited slow shrinkage in volume, an increase in strength, and a decrease in elongation at break. In terms of time, the reduction of bound water stage lasted the longest, and its degree of decrease determined the service life of the PVA hydrogels.

During the final aging stage, with the depletion of residual bound water, the dense material structure and excessive crystalline cross-linking points caused the cross-linking network to become rigid [48], restricting the movement of molecular chains. Therefore, the brittle behavior of the material was apparent.

Similar phenomena and analyses have been observed and corroborated by studies on other hydrogels. Sekine et al. [52] prepared chemically cross-linked PDMAA hydrogels and found that the process of dehydration occurred largely in three stages. The first stage involved the evaporation of free water, during which the polymer network contracted with the evaporation of water. In the second stage, as the bound water decreased, the polymer underwent a glassy “tough-brittle” transition. In the final stage, the remaining bound water slowly evaporated from the glassy dried gel state [53]. However, unlike the chemically cross-linked hydrogels, our research focused on the characteristics of PVA hydrogels, which rely on physical cross-linking through hydrogen bonding and crystallization. Through performance tests such as crystallinity tests, we concluded that the aging of physically cross-linked hydrogels also exhibits three similar stages.

According to the above analysis, the loss of moisture is a primary factor affecting the performance and lifespan of PVA hydrogels. As shown in Figure 10, the reduction of the bound water stage lasts the longest, and its degree of decrease determines the service lifespan of PVA hydrogels. Therefore, the amount of bound water can serve as a crucial indicator for assessing the aging lifespan, and monitoring the bound water content to indirectly evaluate the service life of hydrogels may represent a significant direction for future development. To improve the lifespan, applying hydrophobic coatings on the surface of hydrogels to prepare anti-desiccation modified hydrogels may be an effective methodology [54]. Additionally, future research should focus on developing strategies to prevent the loss of bound water. For instance, the hydrogen bond strength between bound water and molecular chains can be enhanced from a technological standpoint, and molecular engineering techniques can be implemented to regulate these hydrogen bonds [55]. Moreover, PVA hydrogels have the potential to be used in humid and underwater environments because of the slow loss of water in wet environments.

As research into the aging mechanisms of PVA hydrogels progresses, it will be essential to employ more characterization methods to deepen our understanding, which will ultimately contribute to prolonging material lifespan and enhancing potential applications. For instance, to investigate the state of the cross-linked network, DMA and NMR tests can provide reference data for changes in cross-link density and molecular chain segments. TEM experiments can be combined with XRD testing to correlate and validate findings, offering support and deeper insights into the crystallization behavior of PVA hydrogels.

## 5. Conclusions

This study investigated the aging process of PVA hydrogels through a high-temperature accelerated aging test conducted at 60 °C for 80 d, along with macroscopic mechanics, microscopic morphology, and microanalysis tests. The specific conclusions are as follows:

(1) According to the moisture loss characteristics, the aging of PVA hydrogels occurred in three stages: reduction of free water, reduction in bound water, and depletion of bound water.
(2) Notably, the reduction in bound water significantly influences the lifespan of PVA hydrogels, with the maximum elongation serving as the aging characterization parameter of mechanical properties. In terms of time, the reduction in the bound water stage lasts the longest, and its degree of decrease determines the service life of PVA hydrogels.
(3) Microscopically, the aging process of PVA hydrogels is controlled by molecular chain spacing, hydrogen bonds between PVA molecules, and the plasticizing effect of water. During the reduction in the free water stage, molecular chain spacing is the dominant mechanism; during the reduction in the bound water stage, hydrogen bonds between PVA molecules and the plasticizing effect of water are the dominant mechanisms; and during the depletion of the bound water stage, the plasticizing effect of water is the dominant mechanism.
(4) The loss of bound water emerged as the primary factor determining the lifespan of PVA hydrogel structural components. To prolong the service life of PVA hydrogels, future research should focus on developing strategies to impede the loss of bound water, such as environmental humidity control and hydrophobic coating.

## Figures and Tables

**Table 1:** Moisture content and volume changes of samples during aging process.

Aging Time (d)	Volume (mm^3^)	Volume Shrinkage Ratio (%)	Moisture Content (%)
0	3628	/	62
20	2163	40.4	40.13
40	1817	49.9	10.65
60	1607	55.7	8.75
80	1121	69.1	0.31

**Table 2:** Crystallinity changes in samples during aging process.

Aging Time (d)	Crystallinity (%)
0	14.6
20	62.4
40	70.5
60	60.0
80	66.6

## Data Availability

The original contributions presented in the study are included in the article; further inquiries can be directed to the corresponding authors.

## References

[1] Zhang Y.S., Khademhosseini A. (2017). Advances in Engineering Hydrogels. Science.

[2] Burdick J.A., Murphy W.L. (2012). Moving from Static to Dynamic Complexity in Hydrogel Design. Nat. Commun..

[3] Zuo X., Zhou Y., Hao K., Liu C., Yu R., Huang A., Wu C., Yang Y. (2024). 3D Printed All-Natural Hydrogels: Flame-Retardant Materials Toward Attaining Green Sustainability. Adv. Sci..

[4] Zeng C., Gu Y., Xie Y., Hu W., Huang M., Liao G., Yang J., Fan Z., Tan R. (2023). Eco-Friendly Preparation of Carbon-Bonded Carbon Fiber Based on Glucose-Polyacrylamide Hydrogel Derived Carbon as Binder. Nanomaterials.

[5] Zhou C., Wu T., Xie X., Song G., Ma X., Mu Q., Huang Z., Liu X., Sun C., Xu W. (2022). Advances and Challenges in Conductive Hydrogels: From Properties to Applications. Eur. Polym. J..

[6] Zhang W., Feng P., Chen J., Sun Z., Zhao B. (2019). Electrically Conductive Hydrogels for Flexible Energy Storage Systems. Prog. Polym. Sci..

[7] Singh R., Veer B. (2018). Hydrogels: Promising Energy Storage Materials. ChemistrySelect.

[8] Hamidi M., Azadi A., Rafiei P. (2008). Hydrogel Nanoparticles in Drug Delivery. Adv. Drug Deliv. Rev..

[9] Sun Y., Nan D., Jin H., Qu X. (2020). Recent Advances of Injectable Hydrogels for Drug Delivery and Tissue Engineering Applications. Polym. Test..

[10] Kaur P., Agrawal R., Pfeffer F.M., Williams R., Bohidar H.B. (2023). Hydrogels in Agriculture: Prospects and Challenges. J. Polym. Environ..

[11] Liu Y., Wang J., Chen H., Cheng D. (2022). Environmentally Friendly Hydrogel: A Review of Classification, Preparation and Application in Agriculture. Sci. Total Environ..

[12] Liu D., Cao Y., Jiang P., Wang Y., Lu Y., Ji Z., Wang X., Liu W. (2023). Tough, Transparent, and Slippery PVA Hydrogel Led by Syneresis. Small.

[13] Adelnia H., Ensandoost R., Shebbrin Moonshi S., Gavgani J.N., Vasafi E.I., Ta H.T. (2022). Freeze/Thawed Polyvinyl Alcohol Hydrogels: Present, Past and Future. Eur. Polym. J..

[14] Kumar A., Han S.S. (2017). PVA-Based Hydrogels for Tissue Engineering: A Review. Int. J. Polym. Mater. Polym. Biomater..

[15] Ergul N.M., Unal S., Kartal I., Kalkandelen C., Ekren N., Kilic O., Chi-Chang L., Gunduz O. (2019). 3D Printing of Chitosan/Poly(Vinyl Alcohol) Hydrogel Containing Synthesized Hydroxyapatite Scaffolds for Hard-Tissue Engineering. Polym. Test..

[16] Xu Q., Hou M., Wang L., Zhang X., Liu L. (2023). Anti-Bacterial, Anti-Freezing Starch/Ionic Liquid/PVA Ion-Conductive Hydrogel with High Performance for Multi-Stimulation Sensitive Responsive Sensors. Chem. Eng. J..

[17] Xue S., Wu Y., Liu G., Guo M., Liu Y., Zhang T., Wang Z. (2021). Hierarchically Reversible Crosslinking Polymeric Hydrogels with Highly Efficient Self-Healing, Robust Mechanical Properties, and Double-Driven Shape Memory Behavior. J. Mater. Chem. A.

[18] Chae A., Murali G., Lee S., Gwak J., Kim S.J., Jeong Y.J., Kang H., Park S., Lee A.S., Koh D. (2023). Highly Oxidation-Resistant and Self-Healable MXene-Based Hydrogels for Wearable Strain Sensor. Adv. Funct. Mater..

[19] Tayefi M., Eesaee M., Hassanipour M., Elkoun S., David E., Nguyen-Tri P. (2023). Recent Progress in the Accelerated Aging and Lifetime Prediction of Elastomers: A Review. Polym. Degrad. Stab..

[20] Qi-heng T., Run-li N. (2012). Thermal Aging Behavior of High Performance Poly(Vinyl Alcohol) Hydrogel. J. Beijing Inst. Technol..

[21] Cao J., Zhao X., Ye L. (2020). Facile Method to Fabricate Superstrong and Tough Poly(Vinyl Alcohol) Hydrogels with High Energy Dissipation. Ind. Eng. Chem. Res..

[22] Chen J., Yang Z., Shi D., Zhou T., Kaneko D., Chen M. (2021). High Strength and Toughness of Double Physically Cross-linked Hydrogels Composed of Polyvinyl Alcohol and Calcium Alginate. J. Appl. Polym. Sci..

[23] Xiong C., Wei F., Li W., Liu P., Wu Y., Dai M., Chen J. (2018). Mechanism of Polyacrylamide Hydrogel Instability on High-Temperature Conditions. ACS Omega.

[24] Law A., Simon L., Lee-Sullivan P. (2008). Effects of Thermal Aging on Isotactic Polypropylene Crystallinity. Polym. Eng. Sci..

[25] Sližová M., Stašek M., Raab M. (2020). Polypropylene after Thirty Years of Storage: Mechanical Proof of Heterogeneous Aging. Polym. J..

[26] Hodge R.M., Bastow T.J., Edward G.H., Simon G.P., Hill A.J. (1996). Free Volume and the Mechanism of Plasticization in Water-Swollen Poly(Vinyl Alcohol). Macromolecules.

[27] Briscoe B., Luckham P., Zhu S. (2000). The Effects of Hydrogen Bonding upon the Viscosity of Aqueous Poly(Vinyl Alcohol) Solutions. Polymer.

[28] Li H., Zhang W., Xu W., Zhang X. (2000). Hydrogen Bonding Governs the Elastic Properties of Poly(Vinyl Alcohol) in Water: Single-Molecule Force Spectroscopic Studies of PVA by AFM. Macromolecules.

[29] Darabi M.A., Khosrozadeh A., Wang Y., Ashammakhi N., Alem H., Erdem A., Chang Q., Xu K., Liu Y., Luo G. (2020). An Alkaline Based Method for Generating Crystalline, Strong, and Shape Memory Polyvinyl Alcohol Biomaterials. Adv. Sci..

[30] Li L., Xu X., Liu L., Song P., Cao Q., Xu Z., Fang Z., Wang H. (2021). Water Governs the Mechanical Properties of Poly(Vinyl Alcohol). Polymer.

[31] Zhang G., Wang J., Liu X., Li M., Chen C., Wang N., Hou X. (2023). Correlation between the Micro-Structure and Macroscopic Mechanical Properties of GAP-Based Propellant during Aging. Polym. Degrad. Stab..

[32] Liu J., Tong X., Luo X., Chen X., Wang T., Xu J. (2023). The Relaxation Behavior of Composite Double-Base Propellants with Various Stabilizer Content under Thermal Aging. Mech. Time-Depend. Mater..

[33] Chen B., Chen Q., Xiao S., Feng J., Zhang X., Wang T. (2021). Giant Negative Thermopower of Ionic Hydrogel by Synergistic Coordination and Hydration Interactions. Sci. Adv..

[34] Mansur H.S., Oréfice R.L., Mansur A.A.P. (2004). Characterization of Poly(Vinyl Alcohol)/Poly(Ethylene Glycol) Hydrogels and PVA-Derived Hybrids by Small-Angle X-Ray Scattering and FTIR Spectroscopy. Polymer.

[35] Scatena L.F., Brown M.G., Richmond G.L. (2001). Water at Hydrophobic Surfaces: Weak Hydrogen Bonding and Strong Orientation Effects. Science.

[36] Mandal S., Dasmahapatra A.K. (2021). Effect of Aging on the Microstructure and Physical Properties of Poly(Vinyl Alcohol) Hydrogel. J. Polym. Res..

[37] Shi L., Han Q. (2018). Molecular Dynamics Study of Deformation Mechanisms of Poly(Vinyl Alcohol) Hydrogel. Mol. Simul..

[38] Pavia D.L., Lampman G.M., Kriz G.S., Vyvyan J.R. (2008). Introduction to Spectroscopy.

[39] Kudo K., Ishida J., Syuu G., Sekine Y., Ikeda-Fukazawa T. (2014). Structural Changes of Water in Poly(Vinyl Alcohol) Hydrogel during Dehydration. J. Chem. Phys..

[40] Bercea M., Bibire E.-L., Morariu S., Teodorescu M., Carja G. (2015). pH Influence on Rheological and Structural Properties of Chitosan/Poly(Vinyl Alcohol)/Layered Double Hydroxide Composites. Eur. Polym. J..

[41] Peppas N.A., Merrill E.W. (1976). Differential Scanning Calorimetry of Crystallized PVA Hydrogels. J. Appl. Polym. Sci..

[42] Lee J., Jin Lee K., Jang J. (2008). Effect of Silica Nanofillers on Isothermal Crystallization of Poly(Vinyl Alcohol): In-Situ ATR-FTIR Study. Polym. Test..

[43] Holland B.J., Hay J.N. (2001). The Thermal Degradation of Poly(Vinyl Alcohol). Polymer.

[44] Sarma S., Datta P. (2010). Characteristics of Poly(Vinyl Alcohol)/Lead Sulphide Quantum Dot Device. Nanosci. Nanotechnol. Lett..

[45] Chandrakala H.N., Ramaraj B., Shivakumaraiah, Siddaramaiah (2014). Optical Properties and Structural Characteristics of Zinc Oxidecerium Oxide Doped Polyvinyl Alcohol Films. J. Alloys Compd..

[46] Peng M., Xiao G., Tang X., Zhou Y. (2014). Hydrogen-Bonding Assembly of Rigid-Rod Poly(*p*-Sulfophenylene Terephthalamide) and Flexible-Chain Poly(Vinyl Alcohol) for Transparent, Strong, and Tough Molecular Composites. Macromolecules.

[47] Patterson A.L. (1939). The Scherrer Formula for X-ray Particle Size Determination. Phys. Rev..

[48] Kim J., Zhang G., Shi M., Suo Z. (2021). Fracture, Fatigue, and Friction of Polymers in Which Entanglements Greatly Outnumber Cross-Links. Science.

[49] Xu S., Zhou Z., Liu Z., Sharma P. (2023). Concurrent Stiffening and Softening in Hydrogels under Dehydration. Sci. Adv..

[50] Assender H.E., Windle A.H. (1998). Crystallinity in Poly(Vinyl Alcohol). 1. An X-ray Diffraction Study of Atactic PVOH. Polymer.

[51] Chen Y., Li J., Lu J., Ding M., Chen Y. (2022). Synthesis and Properties of Poly(Vinyl Alcohol) Hydrogels with High Strength and Toughness. Polym. Test..

[52] Sekine Y., Ikeda-Fukazawa T. (2009). Structural Changes of Water in a Hydrogel during Dehydration. J. Chem. Phys..

[53] Naohara R., Narita K., Ikeda-Fukazawa T. (2017). Change in Hydrogen Bonding Structures of a Hydrogel with Dehydration. Chem. Phys. Lett..

[54] Zhu T., Jiang C., Wang M., Zhu C., Zhao N., Xu J. (2021). Skin-Inspired Double-Hydrophobic-Coating Encapsulated Hydrogels with Enhanced Water Retention Capacity. Adv. Funct. Mater..

[55] Deng Y., Zhang Q., Qu D.-H. (2023). Emerging Hydrogen-Bond Design for High-Performance Dynamic Polymeric Materials. ACS Mater. Lett..

